# Optimizing vestibular implant electrode positioning using fluoroscopy and intraoperative CT imaging

**DOI:** 10.1007/s00405-023-08428-5

**Published:** 2024-01-05

**Authors:** Elke Loos, Joost J. A. Stultiens, Benjamin Volpe, Bernd L. Vermorken, Stan C. J. Van Boxel, Elke M. J. Devocht, Marc van Hoof, Alinda A. Postma, Nils Guinand, Angelica Pérez-Fornos, Vincent Van Rompaey, Sam Denys, Christian Desloovere, Nicolas Verhaert, Raymond van de Berg

**Affiliations:** 1 Department of Otorhinolaryngology-Head and Neck Surgery, Faculty of Health Medicine and Life Sciences, School for Mental Health and Neuroscience (MHeNS), Maastricht University Medical Center, Maastricht, The Netherlands; 2 Department of Otorhinolaryngology-Head and Neck Surgery, University Hospitals Leuven, Leuven, Belgium; 3 Department of Neurosciences, Research Group Experimental Oto-Rhino-Laryngology (ExpORL), KU Leuven, University of Leuven, Herestraat 49, 3000 Leuven, Belgium; 4 Department of Radiology and Nuclear Medicine, Faculty of Health Medicine and Life Sciences, School for Mental Health and Neuroscience (MHeNS), Maastricht University Medical Center, Maastricht, The Netherlands; 5 Service of Otorhinolaryngology Head and Neck Surgery, Department of Clinical Neurosciences, Geneva University Hospitals, Geneva, Switzerland; 6 Faculty of Medicine and Health Sciences, University of Antwerp, Antwerp, Belgium; 7 Department of Otorhinolaryngology and Head and Neck Surgery, Antwerp University Hospital, Edegem, Belgium

**Keywords:** Vestibular implant, Bilateral vestibulopathy, Imaging, Fluoroscopy, Vestibular dysfunction

## Abstract

**Purpose:**

Vestibular implant electrode positioning close to the afferent nerve fibers is considered to be key for effective and selective electrical stimulation. However, accurate positioning of vestibular implant electrodes inside the semicircular canal ampullae is challenging due to the inability to visualize the target during the surgical procedure. This study investigates the accuracy of a new surgical protocol with real-time fluoroscopy and intraoperative CT imaging, which facilitates electrode positioning during vestibular implant surgery.

**Methods:**

Single-center case-controlled cohort study with a historic control group at a tertiary referral center. Patients were implanted with a vestibulocochlear implant, using a combination of intraoperative fluoroscopy and cone beam CT imaging. The control group consisted of five patients who were previously implanted with the former implant prototype, without the use of intraoperative imaging. Electrode positioning was analyzed postoperatively with a high-resolution CT scan using 3D slicer software. The result was defined as accurate if the electrode position was within 1.5 mm of the center of the ampulla.

**Results:**

With the new imaging protocol, all electrodes could be positioned within a 1.5 mm range of the center of the ampulla. The accuracy was significantly higher in the study group with intraoperative imaging (21/21 electrodes) compared to the control group without intraoperative imaging (10/15 electrodes), (*p* = 0.008).

**Conclusion:**

The combined use of intraoperative fluoroscopy and CT imaging during vestibular implantation can improve the accuracy of electrode positioning. This might lead to better vestibular implant performance.

## Introduction

Bilateral vestibulopathy is a debilitating disorder, which can lead to a broad spectrum of symptoms like postural instability, distorted vision during head movements (oscillopsia), and impairment of spatial orientation [1, 2]. This can strongly impact the quality of life, as it can lead to a decrease in physical activity and social functioning. It can also increase the risk of falling [3–6]. Unfortunately, current treatment options such as physiotherapy or vibrotactile feedback [7], cannot sufficiently restore functionality in most patients [8]. Therefore, investigational vestibular implants were developed to artificially restore vestibular function using electrical currents delivered to the vestibular nerve branches [9]. These devices are analogous to cochlear implants, which are used to restore hearing. The investigational vestibular implant used in this study uses motion sensors to capture motion. This motion information is transferred to electrodes implanted near the ampullary branches of the vestibular nerve [10]. Results of vestibular implantation are promising: electrically evoked vestibulo-ocular reflexes can be generated, and it is possible to (partially) restore vestibular function in a broad head movement frequency spectrum [11, 12]. Additionally, functional benefits were previously demonstrated by improving dynamic visual acuity, controlling postural responses, and increasing quality of life [13–15].

Two approaches for electrode positioning have been used by the Geneva-Maastricht group. The intralabyrinthine technique is most frequently used [16]. In this technique, the electrodes are inserted into the semicircular canals, with the aim of placing the electrode contacts in the ampullae. First, the semicircular canals are bluelined and fenestrated. Subsequently, the electrode leads are inserted ‘blindly’, i.e. without sufficient intraoperative feedback about the final electrode position (since in practice, the bony capsule of the labyrinth impedes visualization of the inserted part of the electrode lead). As a result, the electrode position can be suboptimal with a position not always close to the ampullary nerves. This might compromise neural activation and efficacy of stimulation [17].

Intraoperative imaging could help to improve electrode positioning when using the intralabyrinthine approach. For example, conventional fluoroscopy would allow for a dynamic real-time assessment of electrode positioning. A study with cadaveric human heads already demonstrated the feasibility and value of fluoroscopy for vestibular electrode positioning [18]. Electrodes could be positioned significantly closer to their target, but it remained challenging to sufficiently visualize all semicircular ampullae at the same time due to the three-dimensional anatomy and the overprojection of different structures. Providentially, modern-day fluoroscopy C-arms can often perform a variant of cone beam CT imaging. Therefore, combining fluoroscopy with CT to validate the electrode position adds three-dimensional information, mitigating potential pitfalls of fluoroscopy.

The primary objective of this study was to evaluate the accuracy of vestibular electrode insertions, using a combination of intraoperative fluoroscopy and CT imaging.

## Methods

### Patients

In this case-controlled study, seven patients with bilateral vestibulopathy and sensorineural hearing loss eligible for vestibulocochlear implantation were selected. This study was part of the VertiGO! Trial: a single-center clinical trial at the Maastricht University Medical Center + , in cooperation with Geneva University Hospitals. The full set of in- and exclusion criteria can be found in the study protocol (clinicaltrials.gov (NCT04918745)). The control group consisted of five patients who were previously implanted with a former VCI prototype without intraoperative imaging [19].

### Surgical setup and imaging

All patients were implanted with a VCI containing a cochlear lead with nine electrodes and three vestibular electrode leads (Med-El GmbH, Innsbruck, Austria). All vestibular electrodes contained one ball contact at the tip with a diameter of 0.5 mm. The intralabyrinthine technique was used [16]. The three semicircular canals were fenestrated after performing a cortical mastoidectomy and bluelining of the canals. In the procedure previously followed for the control group, the electrodes were then inserted and positioned without the use of intraoperative imaging techniques.

In the patients of the current trial, the electrodes were first inserted into the semicircular canals without imaging. After that, fluoroscopy was performed (Ziehm Vision RFD 3D, Ziehm Imaging GmbH, Nürnberg, Germany; or CIOS Spin, or Siemens Healthcare GmbH, Erlangen, Germany) to visualize the semicircular canal ampullae and electrodes. The C-arm was arranged in a modified Stenvers position [20]. From this position, the C-arm was slightly adjusted to optimize the visualization of the ampullae and electrodes. Using fluoroscopy, the electrode position was then adjusted to be closer to the center of the ampulla. This way, radiation exposure was minimized to only the visualization and repositioning procedure.

The center of the ampulla was chosen as the target area since the ampulla is identifiable using fluoroscopy and its center is close to the sensorineural epithelium. Other structures such as the epithelium and nerve entrance inside of the semicircular canals cannot be precisely defined with current imaging techniques.

After obtaining a correct position based on the consensus of the radiologist and ENT surgeon (using fluoroscopy), the same C-arm was used to obtain an intraoperative cone beam CT scan (Ziehm Vision RFD 3D, slice thickness 0.5 mm, or Siemens CIOS Spin, slice thickness 0.4 mm). This CT scan was then fused intraoperatively[21] with a preoperative CT scan (SIEMENS SOMATOM Definition AS, slice thickness 0.4 mm), to further evaluate electrode positioning. This was performed using the protocol described by Dees et al. [21] in 3D slicer software (version 5) [22]. This implies that scans were manually aligned, followed by the application of the BRAINSFit registration algorithm [23].

**Fig. 1:**
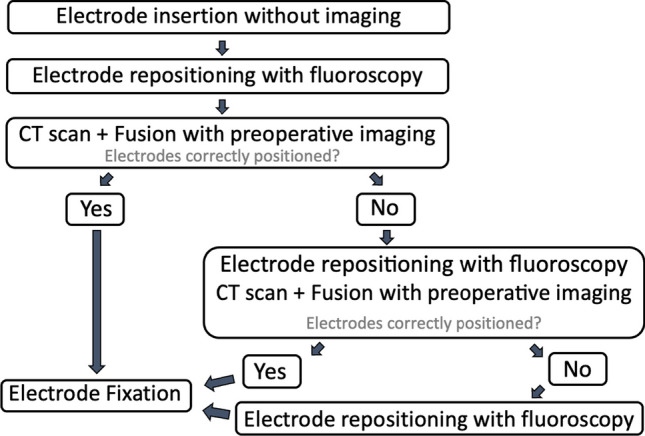
Intraoperative imaging protocol

The need for further repositioning of the electrodes was again evaluated by a neuro-radiologist and the surgeons in consensus. If the position was deemed suboptimal, the fluoroscopy-guided repositioning procedure was repeated and adjustments were once more evaluated with intraoperative CT imaging when indicated (Fig. 1).

All patients received a cone-beam CT (i-CAT Next Generation, Imaging Sciences International, Hatfield, PA, USA, slice thickness 0.2 mm) approximately one week after surgery to visualize and quantify the final electrode position.

### Data analysis

**Fig. 2:**
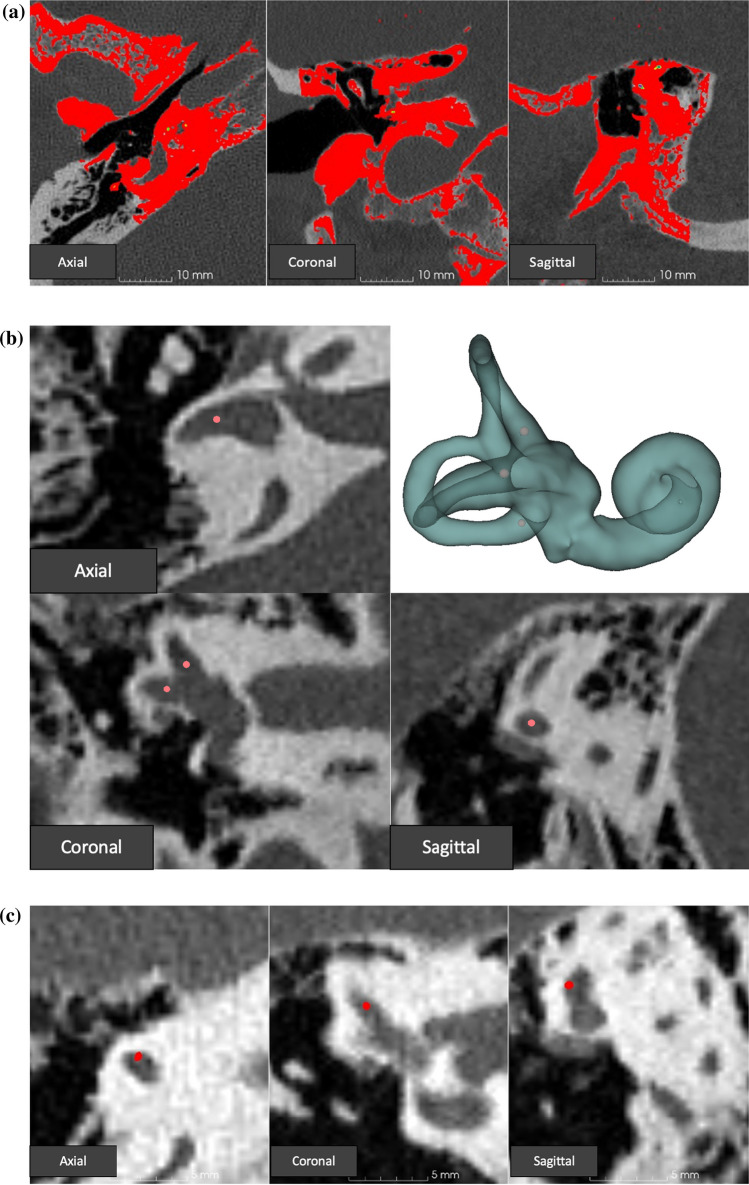
The imaging analysis process (right ear). **A** Manual fusion of the preoperative CT with the postoperative CT in the axial, coronal, and sagittal plane (left to right). Both images are projected in the same window with the postoperative image in front of the preoperative image. The postoperative image is colored (red) to make differences in position more clear. The position of the postoperative CT scan is manually adjusted until both scans are aligned. Next, the BRAINSFit algorithm [23] fuses the images even more precisely. **B** The inner ear is segmented on the preoperative CT scan in the axial, coronal, and sagittal planes. Next, a 3D rendering of the segmentation is created. Consequently, the center of the ampullae of all three canals is defined based on the 3 axes of the CT imaging as well as the 3D segmentation. A fiducial is placed at the defined centers (pink dot). 2C: The fiducials at the center of the ampullae are made invisible. Subsequently, the intensity of the postoperative CT scan (red scan of **A**) is adjusted until only the ball contact, which has the highest intensity (red color) is visible in all 3 planes (axial, coronal, and sagittal). A fiducial, visually enlarged to the same size, is placed in this location

For each vestibular electrode, the distance was measured between the center of the electrode contact and the center of the ampulla of the semicircular canal, as follows. The different steps of distance measurements are demonstrated in Fig. 2. Intra- and postoperative images were fused post hoc with the preoperative CT scan, using the protocol described by Dees et al. [21] in 3D slicer software (version 5) [22]. Subsequently, the semicircular canals were segmented on the preoperative CT scan, and 3D models were rendered. After the segmentation, the centers of the ampullae were determined manually by two investigators on the preoperative CT scan without visualizing the intra- or postoperative scans, to avoid bias. Once the center was defined, a fiducial was placed. Next, the intensity threshold of the intra- and postoperative CT scans was lowered, until the visual diameter of each electrode contact became approximately 0.5 mm, which was equal to the size of the ball contact of the vestibular implant electrodes. The electrode was then marked with a fiducial of the same size (0.5 mm). This was done manually by two investigators independently. Because of the previous fusion of the CT scans, the 3D Euclidean distances could be calculated between the marked electrodes and the center of the ampullae.

The locations of the electrodes could be clearly identified, but in order to lower the measurement error of determining the center of the ampullae, this position was defined 10 times for each canal (blinded for the intra- and postoperative scans, and for the previous fiducials) and the mean of these measurements was used. Additionally, to evaluate the interobserver variability of our measurements, in the first four patients of the study group, those 10 measurements were performed by two researchers separately and blinded (EL and JS). A two-way random-effects model of the intraclass correlation coefficient (ICC) (*k* = 2)) [24] was then performed to rate interobserver variability.

As a primary outcome measure, the accuracy of intraoperative fluoroscopy combined with CT imaging for vestibular electrode insertion was determined. This was investigated by comparing the distances from the electrode tip to the center of the ampulla, between the patients implanted with the use of intraoperative imaging and the control group. Measurements were performed on postoperative CT images. The postoperative CT images were used for this outcome measure, as postoperative CT scans had the best resolution to define the electrode position, and because no intraoperative imaging was available for the control group. The outcome was defined as accurate if the electrode position was within 1.5 mm of the center of the ampulla, as described by Stultiens et al. [25]. This distance was chosen because the average maximal diameter of the ampullae of semicircular canals is around 1.90 mm, with a mean length of 2.46 mm for the posterior canal, 2.30 mm for the superior canal and 2.32 mm for the lateral canal [26]. However, there is a very high interindividual difference with ranges of > 1 mm in length and > 0.5 mm in diameter.

As a secondary outcome measure, the difference between the first intraoperative CT scan (after the first fluoroscopic repositioning) and the last intraoperative CT scan (after additional real-time fluoroscopic adjustment of the electrodes) was investigated. This allowed to evaluate the necessity of performing an additional intraoperative CT scan compared to using fluoroscopy alone. Furthermore, the position of the electrodes on the first intraoperative CT was compared with the control group. This way the advantage of positioning using fluoroscopy alone could be simulated.

Statistical analysis was performed using SPSS software (IBM Corp. Released 2015. IBM SPSS Statistics for Mac VA, NY: IBM Corp). Shapiro–Wilk tests were performed to verify whether the data was normally distributed. For our primary outcome measure, unpaired t-tests (normally distributed variables) and Mann–Whitney U tests (skewed distributed variables) were performed to check for significant differences in the distances (distances from the electrodes to the center of the ampullae for all canals together and all types of canals i.e. lateral, superior, or posterior semicircular canal separately) of the experimental and control groups. The chi-square/fisher exact test was used to check whether the proportion of electrodes categorized as being accurately positioned, was significantly different between the two groups of patients.

For the secondary outcome measure, paired t-tests and Wilcoxon signed ranks tests were used for the continuous data, and the Mc Nemar test was used for proportions of electrodes categorized as being accurately positioned or not. A *p*-value less than 0.05 was considered statistically significant.

### Ethical considerations

This study was approved by the local medical ethical committee (NL73492.068.20/ METC 20–087. It is conducted in accordance with the Declaration of Helsinki (2013 amended version).

## Results

### Combining intraoperative fluoroscopy and CT imaging for vestibular electrode placement

**Fig. 3:**
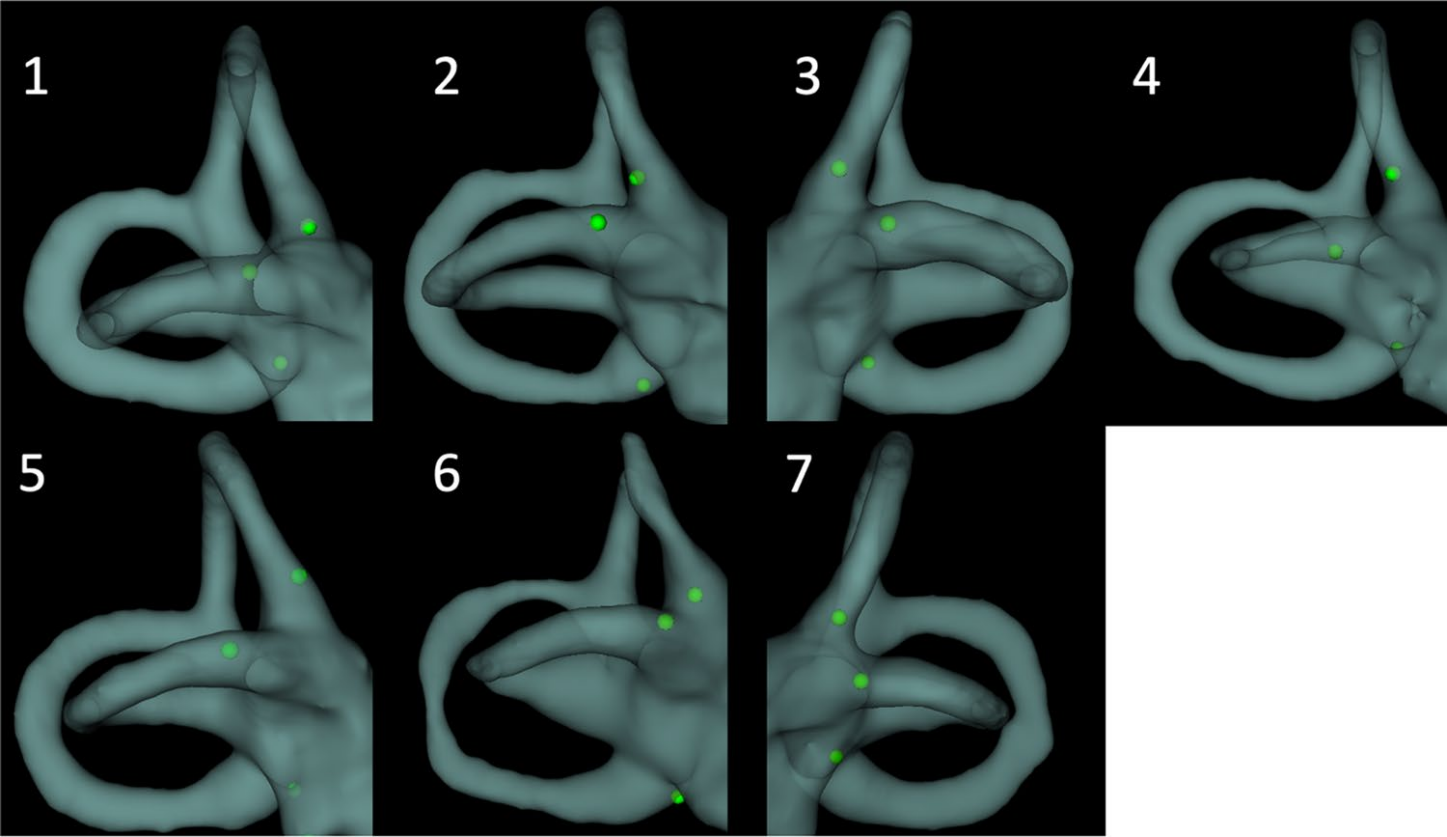
Postoperative 3-dimensional segmentation models of all seven patients implanted with the use of intraoperative fluoroscopy combined with CT. Vestibular electrode contacts are depicted in green

Seven patients (21 electrodes) were implanted using intraoperative fluoroscopy and cone beam CT imaging. Fluoroscopic visualization of the ampulla was possible in all 21 semicircular canals. The interobserver variability of the CT imaging analysis technique was excellent (ICC = 0.96 (95% CI [0.87–0.99])). The median distances from the electrode to the center of the ampulla were 0.87 mm and 0.83 mm for the lateral, 0.86 mm and 0.77 mm for the superior and 1.06 mm and 1.03 mm for the posterior canal in the four patients tested by the two independent researchers (EL and JS respectively). The postoperative distances between the center of the ampullae and the electrodes for every canal are presented in Table 1. The 3D models of the labyrinths are depicted in Fig. 3. The median distances from the electrodes to the center of the ampulla for the lateral, superior, and posterior semicircular canals were 0.89 mm (interquartile range (IQR) 0.64–1.1), 0.85 mm (IQR 0.29–0.90), and 1.11 mm (IQR 0.79–1.35), respectively. The technique was accurate for all 21 electrodes in the seven patients (100%).

**Table 1:** Postoperative and Intermediate Intraoperative distances between the center of the ampullae and the electrodes

Postoperative Distance to center of ampulla (mm)			
	Intraoperative imaging group		
Patient	Lateral SCC	Superior SCC	Posterior SCC
1	0.64	0.85	1.28
2	1.17	0.86	0.83
3	0.64	0.78	0.27
4	0.57	1.00	1.35
5	1.10	0.90	1.38
6	0.89	0.20	1.11
7	0.98	0.29	0.79
Median	0.89	0.85	1.11
Success rate	100%	100%	100%

Patient	Control group		
1	0.62	1.85	0.87
2	3.27	0.99	3.50
3	1.52	0.64	1.41
4	1.00	0.95	1.39
5	2.52	0.63	0.69
Median	1.52	0.95	1.39
Success rate	40%	80%	80%

Intermediate Intraoperative Distance to center of ampulla (mm)			
Patient	Imaging group		
	Lateral SCC	Superior SCC	Posterior SCC
1	0.28	0.48	1.03
2	0.92	0.92	1.32
4	1.16	0.66	2.07
5	1.04	1.05	0.94
6	1.46	0.70	1.11
7	0.99	0.36	2.03
Median	1.015	0.68	1.215
Success rate	100%	100%	67%

On the postoperative imaging, electrodes were positioned using an intraoperative combination of repeated fluoroscopy and CT (Intraoperative imaging group) and without using intraoperative imaging (Control group)*.* In the Intermediate Intraoperative imaging group, electrodes were positioned by one-time use of intraoperative fluoroscopy. *Scc* = *semicircular canal, Success rate* = *number of electrodes* < *1.5 mm from the center of the ampulla*

Data from the control group is also presented in Table 1. The median distances to the center of the ampulla for the lateral, superior, and posterior semicircular canals in this group were 1.52 mm (IQR 0.81–2.90), 0.95 mm (IQR 0.63–1.42), and 1.39 mm (IQR 0.78–2.46) respectively. In 67% (10/15) of the electrodes, a position within 1.5 mm of the center of the ampulla was achieved. Only in one patient (1/5, 20%) all three electrodes were positioned within 1.5 mm of the center of their ampullae.

The proportion of electrodes positioned within 1.5 mm of the center of the ampulla, was significantly higher (*p* = 0.008) in the intraoperative imaging group (21/21 electrodes), compared to the control group without imaging (10/15 electrodes). When comparing median distances from the electrodes to the center of the ampulla between both groups, the electrodes in the study group showed a trend toward closer positioning to the center of the ampulla (*p* = 0.053).

### Fluoroscopy without intraoperative CT imaging

Six out of seven patients (18 electrodes) were included for the evaluation of the necessity of intraoperative CT imaging. One patient (Patient 3) was excluded from this analysis since the intraoperative CT could not sufficiently be fused postoperatively. Specifically, the imaging quality of this intraoperative CT was too low as a result of a procedural error during CT scanning.

In nine of the 18 electrodes (50%), additional fluoroscopic repositioning was performed after the first intraoperative CT scan. The decision to reposition was based on the subjective evaluation of the surgeons in consensus with a radiologist (without objective 3D Euclidean distance measurements). Repositioning was performed twice for the lateral canal electrodes, three times for the superior canal electrodes, and four times for the posterior canal electrodes. Distances between the center of the ampullae and the electrodes after the first intraoperative CT scan can be found in Table 1. The mean distance from the electrodes to the center of the ampullae of all 18 electrodes was 1.03 mm (SD = 0.48) before, and 0.87 mm (SD = 0.27) after fluoroscopic guided repositioning based on the first intraoperative CT scan. There was no significant difference (p = 0.15) between both groups (intraoperative CT before and after repositioning).

**Fig. 4:**
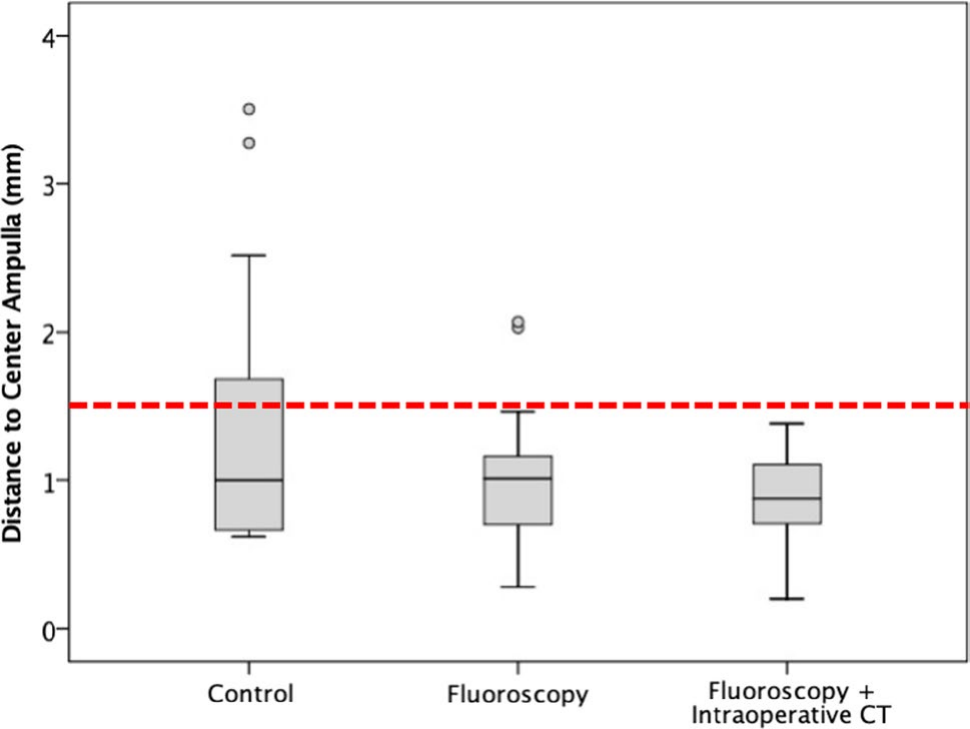
Differences in electrode location between intraoperative fluoroscopy alone and the combination of intraoperative fluoroscopy with CT. The boxplots demonstrate the difference between the distances to the center of the ampulla in the control group without the use of intraoperative imaging (*n* = 15), in the study group after electrode positioning based on intraoperative fluoroscopy alone (*n* = 21), and after electrode positioning based on combining intraoperative fluoroscopy and CT (*n* = 18). The technique was defined as accurate when the electrodes were positioned < 1.5 mm from the center of the ampullae. The red dotted line demonstrates the 1.5 mm limit

The electrode position obtained from the first intraoperative CT was then compared to the control group as if the position of the first intraoperative CT would have been the final position. In other words, as if the electrode positioning was based on fluoroscopy alone. In this scenario, there was no significant difference between both groups regarding the accuracy for obtaining an electrode position within 1.5 mm of the center of the ampulla (p = 0.20). After the first intraoperative CT (electrode position based on fluoroscopy alone), 89% (16/18 electrodes) of the electrodes obtained a position within 1.5 mm of the center of the ampulla. This differed from the control group without intraoperative imaging (67%; 10/15 electrodes) and from combining intraoperative fluoroscopy with CT to position the electrodes (100%; 21/21 electrodes). Figure 4 represents the box plots of all scenarios visually. Fluoroscopy-guided insertion already reduced the number and extent of the outliers and improved the accuracy of electrode positioning compared to blind insertion. The remaining outliers were both located in the posterior canal. However, only when combining intraoperative fluoroscopy and CT imaging for the repositioning, the outliers were completely eliminated.

## Discussion

### Combining intraoperative fluoroscopy and CT imaging for vestibular electrode placement

In this study, the main objective was to investigate the accuracy of using intraoperative fluoroscopy combined with CT imaging, to provide real-time visual guidance during intralabyrinthine vestibular implantation for vestibular implant electrode placement. The intraoperative imaging group showed a significantly higher proportion of electrodes positioned within 1.5 mm of the center of the ampulla (21/21 electrodes), compared to the control group (10/15 electrodes). Therefore, this study demonstrates that the use of intraoperative imaging improves the accuracy of electrode positioning. However, regarding the median distance from the electrodes to the center of the ampulla, there was only a trend towards being shorter when using intraoperative imaging. The difference between the median distance from the electrodes to the center of the ampulla in each group can most likely be attributed to the small sample size of this study. A posthoc power analysis was performed and showed that with the current sample and with an alpha level of 0.05, a power of 0.29 was obtained for statistical differences with a medium effect size of 0.5. This clearly demonstrates that the statistical analysis was underpowered given that one typically aims to achieve a power of 0.8. To obtain this power of 0.8, with the same alpha level and effect size, a sample size of 134 distances (or approximately 54 patients) would be needed. Since vestibular implant research is still in its early stages, obtaining groups of this size would be impossible from a financial, practical, and ethical point of view. Nevertheless, even with this relatively small sample size, a trend could already be observed in favor of the group with intraoperative imaging.

### Fluoroscopy without intraoperative CT imaging for vestibular electrode placement

In this feasibility study, no significant difference was observed between the electrode position on the first intraoperative CT (electrode positioning based on fluoroscopy alone), compared to the last intraoperative CT (electrode positioning based on both fluoroscopy and CT). This might also result from the relatively small sample size. Fluoroscopy alone reduced the number and extent of the outliers, confirming the results of a cadaver study[25]. However, only after repositioning based on the intraoperative CT scan, all outliers (≥ 1.5 mm from the center of the ampulla) were prevented.

Obtaining an accurate technique that makes it possible to insert every electrode within the desired distance from a target, could be very important. After all, correct electrode positioning should improve stimulation efficacy. Electrodes located far from the neural tissue most likely activate fewer nerve fibers and provide less selective stimulation (i.e. current spread to other ampullary nerves or facial nerve). This could lead to less effective stimulation[17]. Nevertheless, the optimal distance of the electrode to the sensory epithelium is currently unknown, considering also the potential for tissue trauma. Also, the spread of excitation patterns in the vestibular organ remains to be fully studied. Therefore, this aspect should be investigated in future studies.

Interestingly, both electrodes that were positioned outside the 1.5 mm range before intraoperative CT imaging and within 1.5 mm after repositioning, were both located inside the posterior canals. The reason for this finding might be explained by the plane in which the fluoroscopic images were visualized. The adjusted modified Stenvers position [20] is most optimal for simultaneous visualization of all three semicircular canals. It is almost perpendicular to the lateral and superior semicircular canals. However, it is not perpendicular to the posterior canal and there is some overprojection of other structures. This makes it more difficult to determine the ampullar region of the posterior semicircular canals on fluoroscopy alone. However, modifications of the C-arm position did not improve fluoroscopic visualization of the posterior ampulla in a previous pilot study of our research group.

In the future, real-time fusion of the preoperative CT scan and the intraoperative fluoroscopic images would be preferred to accurately visualize the ampullae of the semicircular canals. This real-time fusion could save additional intraoperative CT scans and time.

### Limitations

Several limitations were identified in this study. Surgeries in the historic control group were performed by another surgeon than the implantations in the study group, while the vestibular implant design was also adjusted over the years. The implant electrode leads of the historic control group were stiffer, which could also impact the precision of the insertion. However, this would probably only have a minor impact. A prospective study in patients implanted by the same surgeon with an identical implant type would be ideal to accurately study the best imaging protocol for electrode positioning. As stated before, vestibular implant research is still in its early stages, not (yet) allowing for such an extensive study protocol. Secondly, the center of the ampulla was chosen as the target for electrode positioning, although the sensorineural epithelium might be the region of interest for the best functional results[17]. However, it is not possible to visualize the sensory epithelium using intraoperative imaging techniques that are currently available for patients. Segmenting the ampullary nerves on a regular CT scan is both challenging and imprecise, due to the limitations of current imaging resolution. This is especially true for the lateral and superior ampullary nerve fibers. Additionally, these nerves do not have clearly visible entrance points into the ampulla as they enclose the distal part of the ampulla entirely. This limits the correct assessment of the distance to the nerves. This is one of the additional reasons why the center of the ampulla was chosen as the primary target.

## Conclusion

The combined use of intraoperative fluoroscopy and CT imaging during vestibular implantation has the potential to improve the accuracy of electrode positioning. This might lead to better functional results from vestibular implantation.

## Data Availability

The data that support the findings of this study are available in the included tables. Any additional data can be retrieved from the corresponding author, EL, upon reasonable request.
